# High-resolution molecular identification of smalltooth sawfish prey

**DOI:** 10.1038/s41598-019-53931-7

**Published:** 2019-12-04

**Authors:** Taylor L. Hancock, Gregg R. Poulakis, Rachel M. Scharer, S. Gregory Tolley, Hidetoshi Urakawa

**Affiliations:** 1 Department of Ecology and Environmental Studies, The Water School, Florida Gulf Coast University, Fort Myers, Florida 33965 USA; 2 Fish and Wildlife Research Institute, Florida Fish and Wildlife Conservation Commission, Charlotte Harbor Field Laboratory, Port Charlotte, Florida 33954 USA; 3 Department of Marine and Earth Sciences, The Water School, Florida Gulf Coast University, Fort Myers, Florida 33965 USA

**Keywords:** Conservation biology, Ecological networks, Molecular ecology

## Abstract

The foundation of food web analysis is a solid understanding of predator-prey associations. Traditional dietary studies of fishes have been by stomach content analysis. However, these methods are not applicable to Critically Endangered species such as the smalltooth sawfish (*Pristis pectinata*). Previous research using the combination of stable isotope signatures from fin clips and 18S rRNA gene sequencing of fecal samples identified the smalltooth sawfish as piscivorous at low taxonomic resolution. Here, we present a high taxonomic resolution molecular technique for identification of prey using opportunistically acquired fecal samples. To assess potential biases, primer sets of two mitochondrial genes, 12S and 16S rRNA, were used alongside 18S rRNA, which targets a wider spectrum of taxa. In total, 19 fish taxa from 7 orders and 11 families native to the Gulf of Mexico were successfully identified. The sawfish prey comprised diverse taxa, indicating that this species is a generalist piscivore. These findings and the molecular approach used will aid recovery planning for the smalltooth sawfish and have the potential to reveal previously unknown predator-prey associations from a wide range of taxa, especially rare and hard to sample species.

## Introduction

The smalltooth sawfish (*Pristis pectinata*) currently inhabits southwest Florida and the Florida Keys but was once widely distributed on both coasts of the Atlantic Ocean and in the Gulf of Mexico^1^. Decades of human activity, including bycatch mortality in commercial and recreational fishing and loss of red mangrove (*Rhizophora mangle*) shorelines associated with residential and commercial development, have greatly reduced the size of the smalltooth sawfish population^1,2^. Thus, in 2003 the species was listed as endangered under the U.S. Endangered Species Act.

Despite recent expansion of knowledge about the smalltooth sawfish^3^, its feeding ecology is poorly understood. To maximize the effectiveness of ongoing recovery planning, more detailed knowledge of the trophic ecology of smalltooth sawfish is needed to better understand what specific prey they rely on. Traditional dietary studies on piscivores typically involve lethal sampling for stomach content analysis, gastric lavage, and morphological hard part analysis of indigestible prey remains^4–7^. Morphological characterization of prey remains found during gastric lavage or lethal sampling permits conclusions to be drawn about the number and size of fish prey consumed^8,9^; however, lethal sampling is not feasible for endangered species and gastric lavage, though considered non-invasive, may not be a permitted activity for endangered species^10^. Therefore, alternative approaches to identify predator-prey associations are warranted. Recent research using the combination of stable isotope signatures from fin clips and 18S rRNA gene sequencing of fecal samples identified the smalltooth sawfish as piscivorous at low taxonomic resolution^11^. However, a higher taxonomic resolution understanding of this species’ diet is needed to implement effective recovery planning through species-specific management of its prey base, which may include popular commercial and sport fishes.

Here we present a high-resolution molecular method for identification of smalltooth sawfish prey using fecal samples collected over a six-year period. To assess potential biases that might be caused by the selection of primer sets, two mitochondrial genes, 12S and 16S rRNA, were used together with 18S rRNA, a more evolutionarily conserved gene that targets a wider spectrum of taxa^11^. Based on Poulakis and colleagues^11^ previous findings, we expected to find that smalltooth sawfish fed upon rays and various teleost fish species, some of which may be of anthropogenic interest, putting them in direct competition with humans. These data will improve recovery planning for this Critically Endangered species and the analysis technique has the potential to reveal previously unknown predator-prey associations of a wide range of taxa, especially rare and hard to sample species.

## Results

### Smalltooth sawfish fecal sample description

**Figure 1:**
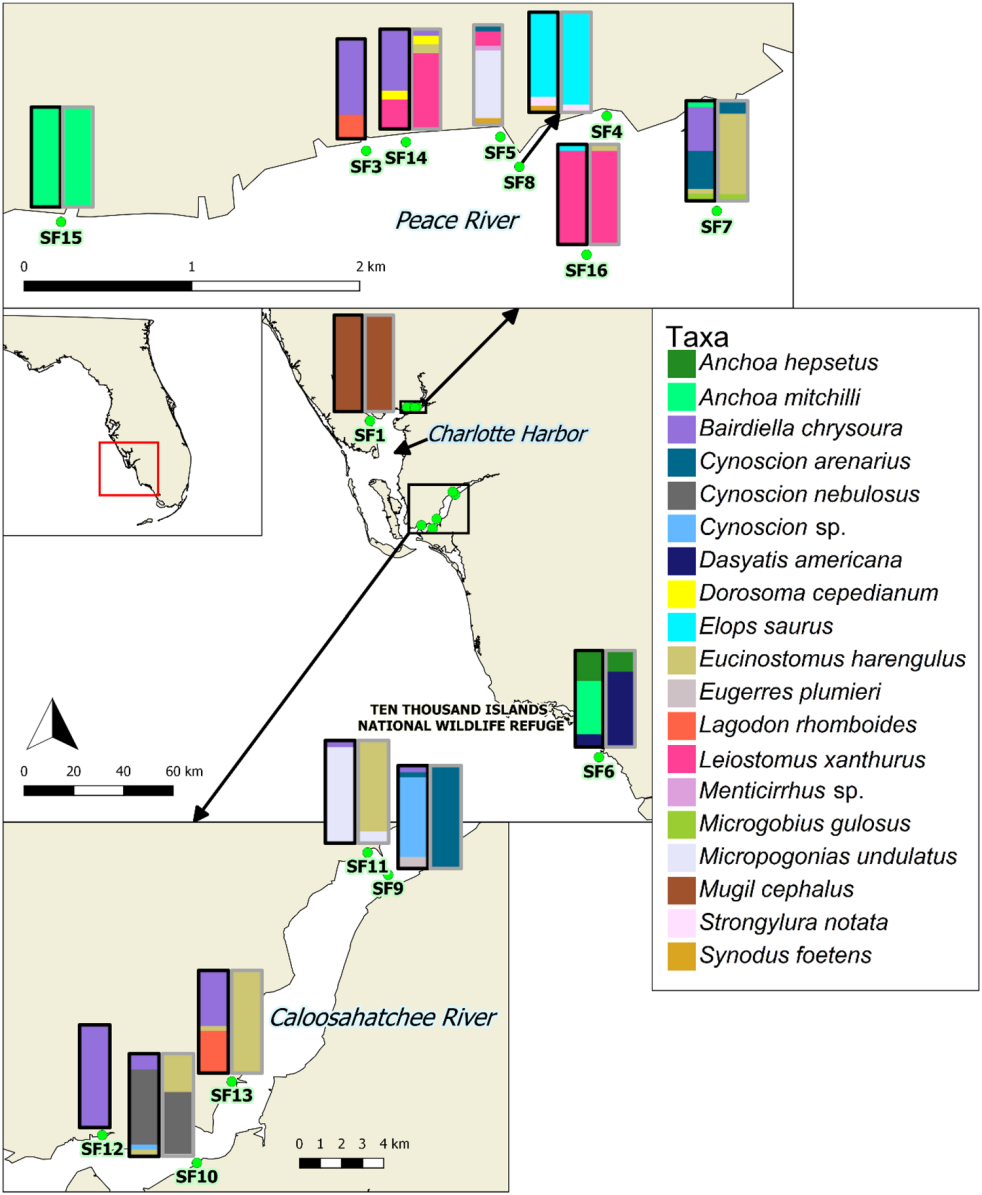
Map of smalltooth sawfish fecal sample collection locations in southwest Florida with stacked bars showing relative composition of fish prey taxa (minimum of 5%), after removal of host sequences. Each 100% stacked bar shows data from mitochondrial 12S (black border) and 16S (grey border) rRNA genes. No fish prey taxa were detected in 16S rRNA gene analysis of samples SF3, SF4, and SF12. DNA sequencing of 12S rRNA gene failed in SF4 and SF5. SF2 did not yield enough DNA for analysis from its extraction. Map generated with QGIS Desktop 2.18 (https://www.qgis.org/en/site/) using data provided by the Florida Fish and Wildlife Conservation Commission-Fish and Wildlife Research Institute.

During field sampling from 2010 through 2015, 16 fecal samples were opportunistically obtained from primarily juvenile (<2500 mm) smalltooth sawfish (780–4355 mm stretch total length; mean = 1398 mm) in a roughly equal proportion of males (*n* = 8) and females (*n* = 7) in southwest Florida from the Charlotte Harbor estuarine system and the Ten Thousand Islands National Wildlife Refuge (Fig. 1; Table 1). DNA was unable to be extracted from one sample due to an insufficient amount of feces.

**Table 1:** Collection data associated with smalltooth sawfish fecal samples.

Sample code^*^	Date	Location	Latitude, longitude	STL^**^ (mm)	Sex
SF1^†^	7/31/2015	Upper Charlotte Harbor	26.91680, −82.18010	4355	F
SF3	7/13/2015	Peace River	26.97085, −82.02990	1020	F
SF4	4/9/2015	Peace River	26.97272, −82.01702	780	M
SF5	5/13/2014	Peace River	26.97160, −82.02272	825	F
SF6^†^	4/19/2015	Ten Thousand Islands	25.70193, −81.35288	1690	F
SF7	5/28/2015	Peace River	26.96660, −82.01118	1020	M
SF8	6/12/2014	Peace River	26.97000, −82.02170	1556	F
SF9	4/23/2015	Caloosahatchee River	26.65053, −81.87252	1447	M
SF10	9/22/2015	Caloosahatchee River	26.52737, −81.95422	1235	M
SF11	5/22/2014	Caloosahatchee River	26.66002, −81.88132	958	F
SF12^‡^	12/21/2010	Caloosahatchee River	26.53923, −81.99467	2026	M
SF13^‡^	6/24/2010	Caloosahatchee River	26.56210, −81.93932	1025	F
SF14^‡^	9/3/2010	Peace River	26.97132, −82.02777	1380	M
SF15	5/1/2013	Peace River	26.96653, −82.04620	835	M
SF16^‡^	4/3/2011	Peace River	26.96465, −82.01815	810	M

^*^SF2 did not yield enough DNA for analysis from its extraction.

^**^STL = stretch (maximum) total length; mean 1398 mm ± SE 232 mm.

^†^Sample obtained from necropsy.

^‡^Sample used in previous 18S rRNA gene analysis (11).

### 18S rRNA gene analysis

**Figure 2:**
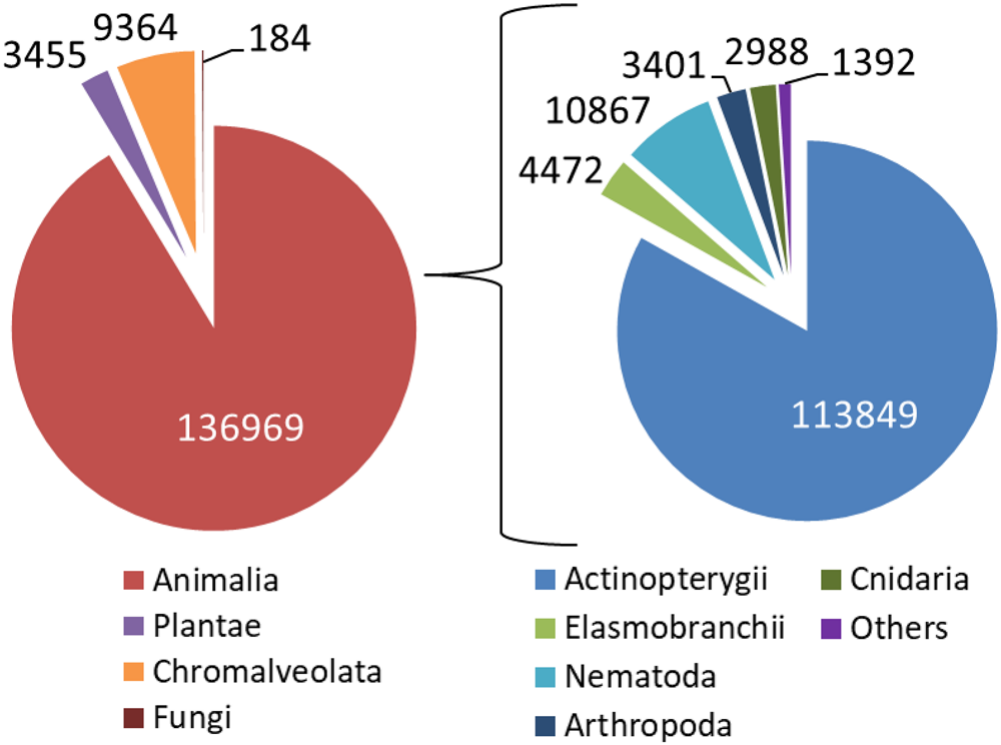
Abundance of normalized 18S rRNA gene sequence reads by Kingdom (left) and composition of Animalia (right) from smalltooth sawfish fecal samples after removal of host sequences. Animalia composed 91.3% of sequence reads (left), with fishes (Actinopterygii and Elasmobranchii) composing 86.4% of Animalia sequence reads (right). Others includes Mollusca and Platyhelminthes.

**Figure 3:**
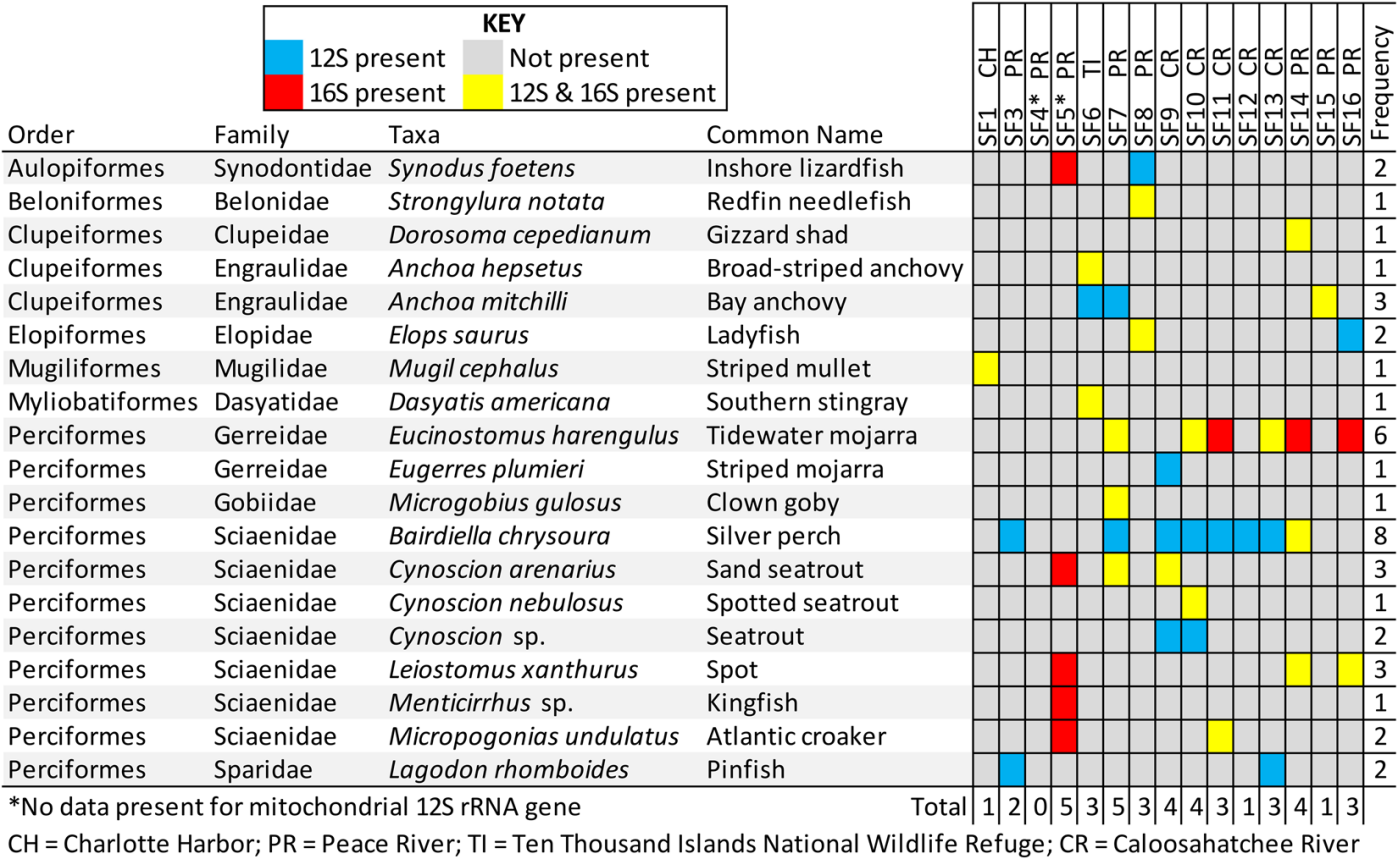
Comparison of fish prey taxa detection in smalltooth sawfish fecal samples using mitochondrial 12S and 16S rRNA gene sequences. Sample locations indicated next to sample code (see footnote). SF2 did not yield enough DNA for analysis from its extraction.

The mean number of analyzed reads for each sample was 110,843 ± 39,647 (±SE; Supplementary Table S1). After normalization (scaled to 10,000 reads) and subsequent removal of host sequences (i.e., smalltooth sawfish), four Kingdoms were identified: Animalia (91.3%), Chromalveolata (6.2%), Plantae (2.4%), and Fungi (0.1%) (Fig. 2). All non-animal taxa were microscopic and considered to be of sediment or ambient water origin. Within Animalia, fishes comprised the majority of sequences (Actinopterygii: 83.1%; Elasmobranchii: 3.3%). In the majority of samples, Arthropoda sequences made up <1% of Animalia sequences; however, considerable numbers were identified in one sample, SF4, with 99.2% of Animalia sequences and 100% of Arthropoda sequences identified as penaeid shrimp (Supplementary Table S2), with 91.5% of these sequences exhibiting 99% similarity to reference sequences (*Litopenaeus setiferus*: JX403844.1; *Farfantepenaeus duorarum*: JX403828.1). SF4 was the smallest individual in our analysis (780 mm) and had no fish prey detected in our mitochondrial 16S rRNA gene analysis (Fig. 3).

### Mitochondrial 12S and 16S rRNA gene analysis

Preliminary results revealed large amounts of unidentified sequences present in our samples and a lack of local fish species reference sequences in GenBank, indicating the need for additional sequencing effort. Thus, we sequenced 24 fish species, including nine orders and 13 families, known to inhabit our sampling area^12^, yielding 16 and 23 additional sequences for the mitochondrial 12S and 16S rRNA genes, respectively (Supplementary Table S3). The high specificity of these sequences allowed closely related species to be distinguished in most cases, which was beneficial in determining the highest resolution (i.e., species level) identifications for Operational Taxonomic Units (OTUs) with Basic Local Alignment Search Tool (BLAST) results that did not meet our identification criterion (98% similarity) or exhibited ties. To provide even more robust analysis, species signature sequences consisting of ~40 bp within 12S and 16S rRNA were identified for 150 and 128 species (Supplementary Table S4), respectively, to conduct manual alignments of unidentified sequences during the final step of our identification protocol. This tag-sequencing strategy attributed to 17.4% and 2.8% of identified 12S and 16S rRNA sequences, respectively (Supplementary Table S5).

**Figure 4:**
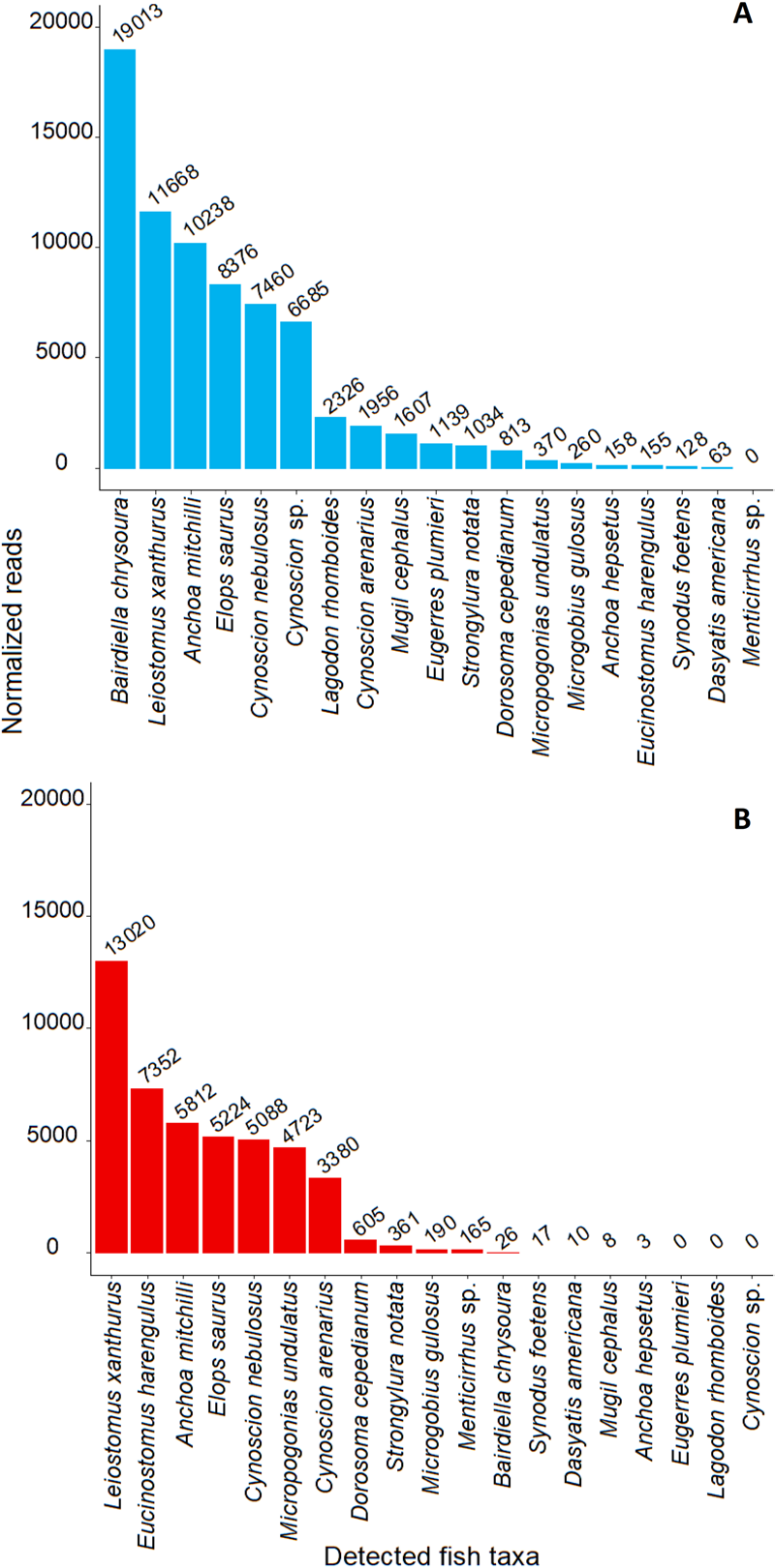
Normalized abundance of detected fish prey taxa in mitochondrial 12S (**A**) and 16S (**B**) rRNA gene sequence reads from smalltooth sawfish fecal samples. In the 12S rRNA gene analysis, 18 taxa were identified, and in the 16S rRNA gene analysis, 16 taxa were identified. The same taxa were included in both graphs for comparative purposes.

The mean (±SE) number of analyzed reads for all samples was 8,963 ± 1,074. Two samples (SF4, SF5) failed to sequence for 12S rRNA analysis due to unsuccessful PCR amplicon generation. After removal of host sequences, our analysis identified 91.3 ± 1.8% of 12S rRNA and 88.9 ± 4.9% of 16S rRNA gene sequence reads at a minimum of genus (Table 2). Of 19 total fish taxa detected, including 7 orders and 11 families, 17 were identified to species, with a mean of 15.1 ± 3.2 OTUs and 2.3 ± 0.3 fishes identified in 12S rRNA fecal samples and 6.8 ± 2.3 OTUs and 1.7 ± 0.4 fishes identified in 16S rRNA fecal samples. Silver perch (*Bairdiella chrysoura*), bay anchovy (*Anchoa mitchilli*), tidewater mojarra (*Eucinostomus harengulus*), spotted seatrout (*Cynoscion nebulosus*), ladyfish (*Elops saurus*), and spot (*Leiostomus xanthurus*) were most prevalent in our analysis (Figs. 3 and 4; Supplementary Table S6). For each gene, Bray Curtis similarity indices were calculated after removing individuals in which no prey taxa were detected. A hierarchical clustering based on these results showed that similar river originated samples (i.e., Peace River and Caloosahatchee River) exhibited similarities and clustered together (Supplementary Fig. S1).

**Table 2:** Analyzed sequence reads of mitochondrial 12S and 16S rRNA genes and relative abundance of host and fish prey sequences.

Sample code	Analyzed sequence reads		Smalltooth sawfish sequences (%)		Fish prey sequence identified (%)^†^	
	12S	16S	12S	16S	12S	16S
SF1	5456	21202	82.3	99.9	91.0	75.0
SF3	2993	3026	11.3	100.0	95.4	0
SF4	ND*	16344	ND	100.0	ND	0
SF5	ND	14967	ND	31.4	ND	90.1
SF6	3675	24865	93.3	99.7	76.7	40.7
SF7	4199	11418	48.8	62.8	89.6	98.3
SF8	5958	12732	2.0	40.8	96.0	94.4
SF9	4783	8607	19.4	70.2	97.3	98.9
SF10	7300	9213	2.3	17.4	94.6	98.1
SF11	9702	7080	95.4	97.6	81.3	91.3
SF12	7371	8584	73.2	100.0	91.0	0
SF13	16437	10169	90.1	98.1	87.5	97.4
SF14	4945	9795	0.5	31.9	90.4	84.4
SF15	10896	4248	0.7	41.3	99.7	99.1
SF16	933	4069	5.3	24.1	97.1	99.1
Total	84648	166319	-	-	-	-
Mean	6511	11088	40.3	67.7	91.3	88.9
±SE	±1112	±1593	±11.3	±8.6	±1.8	±4.9

^*^ND = no data.

^†^After removal of host sequences.

The 16S rRNA primer set appeared to be less sensitive than the 12S rRNA primer set when comparing samples sequenced for both genes. In 16S rRNA analysis, only three (all tidewater mojarra) of 26 identification instances were not corroborated by the other gene, whereas for 12S rRNA, 16 of 34 identification instances were not corroborated. Additionally, the 16S rRNA primer set was unable to identify any fish taxa for two samples (SF3, SF12), and striped mojarra (*Eugerres plumieri*) and pinfish (*Lagodon rhomboides*) were only identified using the 12S rRNA primer set (Fig. 3). While two seatrout species, sand seatrout (*C*. *arenarius*) and spotted seatrout (*C*. *nebulosus*), were identified in both analyses, species were not always distinguishable in our 12S rRNA analysis with some identifications relegated to *Cynoscion* sp., indicating that multiple primer sets may be desirable to overcome potential biases.

## Discussion

Traditional dietary studies of fishes have mainly focused on stomach content analysis, using invasive methods that cannot be used to study Critically Endangered species such as the smalltooth sawfish. DNA-based prey identification can overcome many of these limitations, and a variety of molecular methods have been developed over the past decade to enable the investigation of feeding ecology at unprecedented resolution^13–16^. Moreover, even heavily digested prey remains can be identified by these methods to track trophic links^17^. Molecular techniques have been used to assess the diet of piscivores with a focus on marine predators such as pinnipeds^18^, squids^19^, and seabirds^20^; however, this is the first study achieving species level resolution for elasmobranchs. In addition, there has been limited application of these techniques for studying the feeding ecology of fishes more generally^21^.

In the present study, we combined multiple molecular markers to identify fish prey to species. Mitochondrial 12S and 16S rRNA genes were used to target fishes, which were previously identified as the largest fraction of smalltooth sawfish prey taxa via the 18S rRNA gene^11^. Using a set of high-resolution mitochondrial genes expanded our results by accounting for differences between the two primer sets, with samples exhibiting variation in taxa detection and proportions between them. An example of the need for multiple genes occurred with silver perch and tidewater mojarra which were often detected together but exhibited drastic differences in sequence proportions between genes. In some samples, the silver perch was only detected by 12S rRNA while tidewater mojarra was only detected using 16S rRNA. At first, we thought this indicated an error in our analysis or sample preparation for Sanger sequencing, but after comparing identified query sequences of both species for both genes, our sequences of both species, and reference sequences of congeners, all sequences identified as one or the other species via multiple sequence alignment. This process indicated that both sequences belong to these two species, were well differentiated from each other, were similar to other species within their respective genera, and met our threshold for valid identifications. Without the use of two overlapping high taxonomic resolution primer sets, there would have been undetected or undervalued taxa due to biases of individual primer sets and their respective genes.

We also used species-signature sequences consisting of around 40 bp to refine our data, increasing the number of successfully identified sequences and OTUs. However, in two samples, we were unable to differentiate species in the genus *Cynoscion* for 12S rRNA OTUs. The only additional *Cynoscion* species not identified in the study was the silver seatrout (*C*. *nothus*), which is not known to use the estuary where these samples were collected^12,22^. In one sample, we were unable to differentiate species in the genus *Menticirrhus* for 16S rRNA OTUs. Although our identification protocol identified these as *M*. *littoralis*, this species is not known to use the estuary where this sample was collected^22^, and relevant reference sequences of the two possible other *Menticirrhus* species (i.e., *M*. *americanus* and *M*. *saxatilus*) were absent from DNA databases. Further, Pompanon and colleagues^14^ discussed various potential biases of this new technology that could hamper quantitative questions, such as differences in amplification efficiency or DNA survival during digestion. Because the methodology is progressing rapidly, these problems might be minimized in the near future; however, some issues, such as variation in prey size and the time the prey was consumed, are not easily addressed. For example, we were unable to rule out instances of secondary predation (i.e., consumption of a predator which has consumed the target prey), which has not been addressed in any vertebrate predator^13,23^. These challenges are generally present in feeding ecology analyses using traditional techniques and are not limited to molecular methods^24^.

A high-throughput sequencing approach, able to detect trace amounts of prey, was first used on sawfish as a complementary method to assist studies using stable isotopes, a powerful and widely accepted technique in which trophic levels can be identified, but species level prey identification is impossible^11,25–29^. Poulakis and colleagues^11^ studied the trophic ecology of the smalltooth sawfish in South Florida using a combination of stable isotopes and 18S rRNA gene sequencing of fecal samples, providing evidence that the species feeds primarily on teleost and elasmobranch fishes. In the present study, we replicated and built on this technique with a larger sample size, resulting in successful identification of more prey and reaffirming the previous conclusion that this species is piscivorous. Furthermore, the prey identified comprised diverse taxa (19 fish taxa from 7 orders and 11 families), indicating that this species is a generalist piscivore. Fish prey items were detected in fecal samples from all individuals with the exception of one, which fed primarily on penaeid shrimp, which we speculate was scavenged discarded bait.

The fish prey we identified mirrored the habitats juvenile smalltooth sawfish are known to use within the Charlotte Harbor estuary^30^. Sawfish preyed on species such as the tidewater mojarra that tends to be found along mangrove shorelines, silver perch and spotted seatrout that are typically found on offshore seagrass flats, and bay anchovies and pinfish that are found in both habitats^31^. Acoustic tracking and monitoring in the study area has shown that smalltooth sawfish use all habitats available to them^32,33^, and the present study suggests that they feed on fishes in all of these habitats. Additionally, we did not observe an ontogenetic dietary shift in our data, which is a trait common amongst elasmobranchs^34,35^. No clear differences in prey were observed between juvenile age classes (less than 1 year old: <150 cm STL; greater than 1 year old: >150 cm STL^36^), with fishes such as bay anchovy, ladyfish, and silver perch found in each. A dietary shift may become evident if more samples from larger juveniles and adults could be collected as these age classes are known to use more diverse habitats^3,11^.

Although we know little about sawfish feeding behavior and diet beyond anecdotal reports and previous stable isotope analysis^11^, this study presents multiple new findings, such as the presence of anchovies, one of the smallest fish prey items identified, and the southern stingray, which was anticipated by the 18S rRNA analysis that identified Myliobatiformes in the diet^11^. Recent behavioral experiments have shown captive sawfish feeding with rapid lateral swipes of their rostra in the water column and close to the bottom^37^. Our data suggest that smalltooth sawfish are generalist piscivores that feed on pelagic and benthic fishes.

These findings will aid recovery planning for the smalltooth sawfish and the molecular approach used has the potential to reveal previously unknown predator-prey associations from a wide range of taxa, especially protected species. This study shows that smalltooth sawfish consume a variety of fish prey species found across a diverse range of estuarine habitats. Thus, maintaining healthy estuaries, including healthy fish populations, will be important for promoting recovery of the smalltooth sawfish population and should be more effective than species-specific management approaches. Additionally, climate change and associated environmental impacts may destabilize current habitats for the species and alter habitat use, further emphasizing the need for ecosystem level conservation^38^.

## Materials and Methods

### Smalltooth sawfish fecal sample collection and DNA extraction

From 2010 to 2015, 16 fecal samples were opportunistically obtained from primarily juvenile smalltooth sawfish in southwest Florida during ongoing field sampling or from necropsies (Table 1). All samples were stored at −20 °C until further analysis. DNA extractions were performed using the Quick-DNA Fecal/Soil Microbe Kits (Zymo Research, Irvine, CA, USA) according to manufacturer instructions. DNA was unable to be extracted from one sample (SF2) due to an insufficient amount of feces and 15 fecal samples were used in further sequencing analysis. All methods were carried out in accordance with relevant guidelines and regulations.

### High-throughput sequencing

The extracted DNA samples were used for Illumina sequencing to determine the 18S rRNA, mitochondrial 12S and 16S rRNA genes. Amplification of the 18S rRNA gene was conducted using a universal primer pair (TAReuk454FWD1 [CCA GCA SCY GCG GTA ATT CC] and TAReukREV3 [ACT TTC GTT CTT GAT YRA]). We also applied Illumina sequencing to mitochondrial 12S and 16S rRNA genes to detect fish taxa^39^. The mitochondrial 12S rRNA gene was amplified using a universal primer pair for Actinopterygii (Ac12Sf [ACT GGG ATT AGA TAC CCC ACT ATG] and Ac12Sr [GAG AGT GAC GGG CGG TGT]). The mitochondrial 16S rRNA gene was amplified using a universal primer pair for Actinopterygii (Ac16Sf [CCT TTT GCA TCA TGA TTT AGC] and Ac16Sr [CAG GTG GCT GCT TTT AGG C]). The DNA samples were amplified for sequencing in a two-step process. The forward primer was constructed (5′-3′) with the forward Illumina overhang adapter (TCG TCG GCA GCG TCA GAT GTG TAT AAG AGA CAG) added to the forward primer used. The reverse primer was constructed (5′-3′) with the reverse Illumina overhang adapter (GTC TCG TGG GCT CGG AGA TGT GTA TAA GAG ACA G) added to the reverse primer used. Amplifications were performed in a 25 µL reaction with the Qiagen HotStar Taq master mix (Qiagen, Valencia, California, USA), 1 µL of each 5 µM primer, and 1 µL of template. Reactions were performed on the ABI Veriti thermocycler (Applied Biosytems, Carlsbad, California, USA) under the following thermal profile for 18S rRNA: an initial denaturation for 5 min at 95 °C, followed by 35 cycles of denaturation for 30 s at 94 °C, annealing for 1.5 min at 50 °C and ramp up at 0.5 °C per cycle (1.5 min) in the first 10 cycles and the following 25 cycles at 54 °C, and extension for 1 min at 72 °C, with a final extension of 10 min at 72 °C. In 12S and 16S rRNA analyses, we used the same thermal profile, but with a constant annealing temperature of 54 °C for all 35 cycles. The PCR product from the first stage amplification was added to a second PCR based on qualitatively determined concentrations. Primers for the second PCR were designed based on the Illumina Nextera PCR primers as follows: forward - AAT GAT ACG GCG ACC ACC GAG ATC TAC AC [i5index] TCG TCG GCA GCG TC and reverse - CAA GCA GAA GAC GGC ATA CGA GAT [i7index] GTC TCG TGG GCT CGG. The second stage amplification was run the same as the first except with only 10 cycle extensions. For the 18S rRNA gene, the first stage thermal profile used in 12S and 16S rRNA gene amplification was applied for these 10 cycles. Amplicons were visualized with eGels (Life Technologies, Grand Island, New York, USA) and products were pooled equimolar and each pool was size selected in two rounds using SPRIselect reagent (Beckman Coulter, Indianapolis, Indiana, USA) in a 0.75 ratio for both rounds. Size selected pools were then quantified using the Qubit 4 fluorometer (Life Technologies). The final library pool was analyzed on the MiSeq system (Illumina, San Diego, California, USA) using a Miseq reagent kit v3 (Illumina) with pair-end run setting of 2 × 300 flow cell at 10 pM. We ran 12S and 16S rRNA gene amplification on the same Miseq reagent kit, and 18S rRNA gene was run separately. All PCR reactions were run with no-template control; the negative control was also tagged and sequenced along with samples for contamination check purposes. Illumina sequencing was performed at RTL Genomics (Lubbock, Texas, USA).

To analyze the sequence data, forward and reverse reads were taken in FASTQ format and merged using PEAR Illumina paired-end read merger^40^. The formatted FASTQ files were then converted into FASTA-formatted files for subsequent analyses. DNA sequences were clustered into OTUs at 97% similarity using the CD-Hit-Est clustering algorithm^41,42^. OTUs consisting of less than five sequences were considered inconsequential and omitted from further analyses (12S rRNA gene sequences omitted: 2.06 ± 0.23% [mean ± SE]; 16S rRNA: 0.65 ± 0.08%; 18S rRNA: 1.28 ± 0.06%), with the remainder entering our identification protocol using BLAST.

Sequencing error of 12S and 16S rRNA genes was estimated to be 2% by comparing OTU centroid sequences identified as smalltooth sawfish by BLAST and their respective similarity percentages with a reference sequence (GenBank Accession: KP400584) for each gene via multiple sequence alignment using the MUSCLE program within MEGA7 software^43^ to manually check for chimeric sequences (Supplementary Fig. S2). This estimated sequencing error was used in our sequence identification protocol. Sequencing error was not estimated for 18S rRNA gene analysis due to its low associated taxonomic resolution, with the taxonomic class of the highest scoring BLAST similarity result accepted for each OTU. When class was not applicable, the next highest taxonomic level was used. Taxonomy follows Page and colleagues^44^ for fishes and Williams^45^ for crustaceans.

### Mitochondrial 12S and 16S rRNA gene sequence identification protocol

Based on our estimation of sequencing error found in the host, BLAST results with a similarity score ≥98% were accepted for each prey OTU. If this criterion was met and ties were present, OTUs would be moved to the next step of our identification protocol. The remaining OTUs along with those with ties were aligned with ~40 bp species signature sequences identified for all fish species documented in the sample collection area^12^ that either had reference sequences available in GenBank or were sequenced for this study (Supplementary Table S1). This tag-sequencing strategy determined ~40 bp species signature sequences based on available data, with each sequence checked for specificity via BLAST. Exact matches were accepted as an accurate identification. If an exact match was not made with a tied OTU, the lowest shared taxonomic classification between the tied BLAST results was accepted (none were found higher than genus level, and all were *Cynoscion* sp.). All results were checked against available fisheries data^22^, with any dubious identifications relegated to the lowest viable taxonomic classification (none higher than genus level and all were *Menticirrhus* sp.).

### Sanger sequencing for mitochondrial 12S and 16S rRNA genes of potential fish prey

Twenty-four fish species known from our sampling area were sequenced (Fig. 1; Supplementary Table S3). Sixteen species were sequenced for the mitochondrial 12S rRNA gene and 23 species for the mitochondrial 16S rRNA gene using the same primer sets used in the high-throughput sequencing^39^ except for a minor modification in Ac16Sr(C-) [CAG GTG GCT GCT TTT AGG C]). Preparation of sequencing samples was carried out as described previously^11^.

### Data deposit

High-throughput mitochondrial 12S and 16S rRNA, and 18S rRNA gene sequences of smalltooth sawfish fecal samples were deposited in the National Center for Biotechnology Information sequence read archive under accession number SAMN10130720. Mitochondrial 12S and 16S rRNA gene fish sequences were deposited in GenBank under accession numbers MH715297-312 and MH715980-6002, respectively.

## Supplementary information

Supplementary Information
