# Supplementary material for: High-resolution molecular identification of smalltooth sawfish prey
Source: Sci Rep. 2019 Dec 4;9:18307. doi: 10.1038/s41598-019-53931-7 (PMC6892823; doi:10.1038/s41598-019-53931-7)
Supplement: Supplementary file 1 — Supplementary Information [file 41598_2019_53931_MOESM1_ESM.pdf]

High-resolution molecular identification of smalltooth sawfish prey

Taylor L. Hancock[1], Gregg R. Poulakis[2], Rachel M. Scharer[2], S. Gregory Tolley[3], and Hidetoshi, Urakawa[1,*]

[1]Department of Ecology and Environmental Studies, The Water School, Florida Gulf Coast University, Fort Myers, Florida 33965, USA

[2]Fish and Wildlife Research Institute, Florida Fish and Wildlife Conservation Commission, Charlotte Harbor Field Laboratory, Port Charlotte, Florida 33954, USA

[3]Department of Marine and Earth Sciences, The Water School, Florida Gulf Coast University, Fort Myers, Florida 33965, USA

*Corresponding author

Dr. Hidetoshi Urakawa, Ph. D.

Department of Ecology and Environmental Studies, The Water School, Florida Gulf Coast University, Fort Myers, Florida 33965, USA; hurakawa@fgcu.edu.

Supplementary Figure S1.

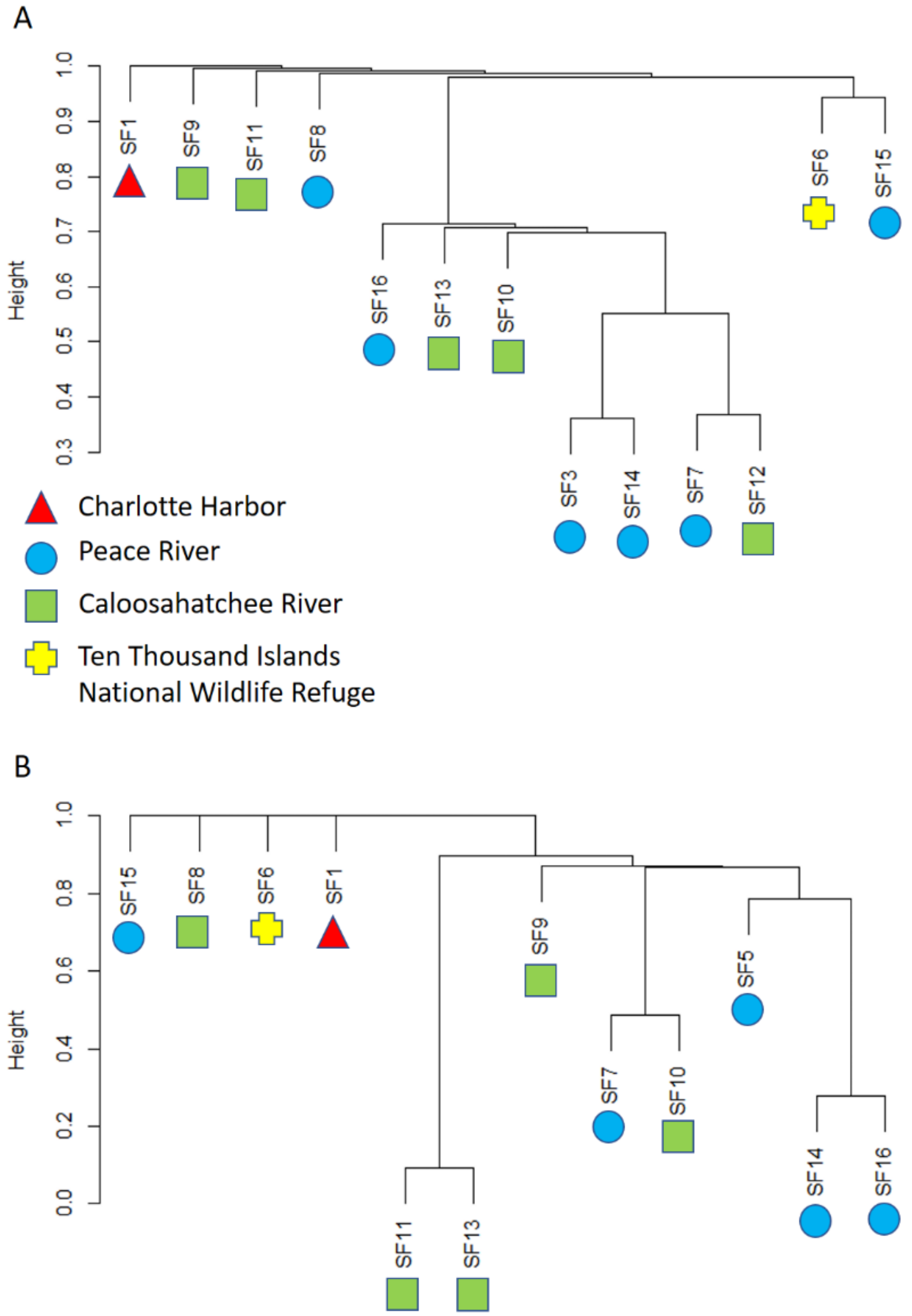

Fig. S1. Hierarchical clustering dendrogram of Bray Curtis similarity analysis of samples for mitochondrial 12S (A) and 16S (B) rRNA genes after removing individuals in which no prey taxa were detected. River originated samples (Peace River and Caloosahatchee River) exhibited some similarities and clustered together.

Supplementary Figure S2.

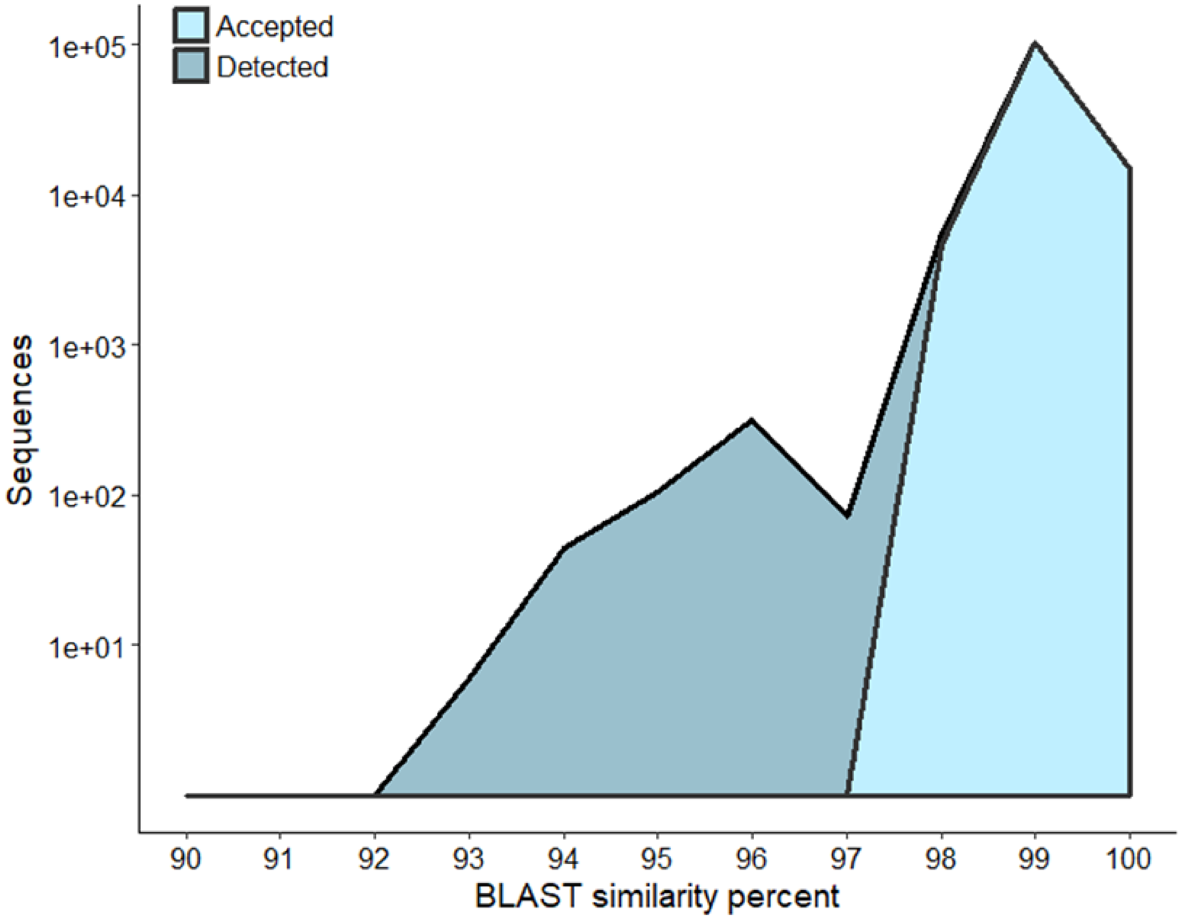

Fig. S2. Estimation of smalltooth sawfish host sequencing errors determined using sequence distributions in Basic Local Alignment Search Tool similarity scores. Distribution of mitochondrial 12S and 16S rRNA sequences identified as smalltooth sawfish are shown as areas (dark blue, errors were detected and rejected; light blue, accepted) on a logarithmic scale with corresponding BLAST similarity scores. These distributions were used to estimate sequencing error, resulting in the conclusion that a BLAST similarity score of ≥ 98% was accurate in our prey identification protocol.

Supplementary Table S1. Relative taxonomic abundance of 18S rRNA gene sequences (%) in smalltooth sawfish fecal samples after removal of host sequences.

Taxa	Classification	SF1	SF3	SF4	SF5	SF6	SF7	SF8	SF9	SF10	SF11	SF12	SF13	SF14	SF15	SF16
Actinopterygii	Class	7.67	96.22	0.47	89.01	48.78	82.71	95.92	97.60	92.43	55.08	44.94	48.42	99.60	95.87	77.20
Amoebozoa	Phylum								0.12							
Anthozoa	Class												18.63	0.03		
Asterales	Order								0.78							
Bacillariophyceae	Phylum								0.10							1.18
Bivalvia	Class								0.12							1.53
Chlorophyceae	Class	22.81	0.06		0.06				0.44					0.06		0.70
Chondrichthyes	Subclass	1.03	1.03		7.17	18.90	6.45	1.68	0.13	6.17	34.76		0.21	0.16	4.04	0.58
Chromadorea	Class	24.72	0.17							1.03		32.58	31.92			0.02
Conoidasida	Class	0.47	0.05		0.32			0.19			10.16	2.81	0.45			16.63
Coscinodiscophyceae	Class	11.55												0.01		
Dinophyceae	Phylum	16.58		19.81	3.31		10.84									
Enoplea	Class											8.43	0.13			
Gastrotricha	Phylum											11.24				
Haptophyceae	Class								0.41							
Hexanauplia	Class														0.03	
Katablepharidophyta	Class			23.07												
Labyrinthulomycetes	Class							0.12								0.04
Malacostraca	Class		2.47	56.65	0.13			0.17		0.33				0.12		
Malpighiales	Order	0.20														
Mammalia	Class															0.82
Maxillopoda	Class	6.87														
Myxosporea	Class	6.19				32.32		1.92		0.04					0.06	1.12
Spirotrichea	Class								0.30							
Poales	Order	0.57												0.02		
Saccharomycetes	Class	1.34														
Streptophyta	Class															0.18
Trematoda	Class												0.24			

Supplementary Table S2. Fish species in sampling area sequenced for mitochondrial 12S and 16S rRNA genes.

Family	*Species*	Common name	Gene(s) sequenced	GenBank Accession #
Belonidae	*Strongylura notata*	Redfin Needlefish	16S	MH716001
Dasyatidae	*Dasyatis americana*	Southern Stingray	12S, 16S	MH715301, MH715984
Dasyatidae	*Dasyatis sabina*	Atlantic Stingray	12S, 16S	MH715302, MH715985
Gerreidae	*Eucinostomus gula*	Silver Jenny	16S	MH715986
Gerreidae	*Eucinostomus harengulus*	Tidewater Mojarra	12S, 16S	MH715303, MH715987
Gerreidae	*Eugerres plumieri*	Striped Mojarra	16S	MH715988
Gobiesocidae	*Gobiesox strumosus*	Skilletfish	16S	MH715989
Gobiidae	*Bathygobius soporator*	Frillfin Goby	12S, 16S	MH715298, MH715981
Gobiidae	*Ctenogobius boleosoma*	Darter Goby	12S, 16S	MH715299, MH715982
Gobiidae	*Ctenogobius smaragdus*	Emerald Goby	12S, 16S	MH715300, MH715983
Gobiidae	*Gobionellus oceanicus*	Highfin Goby	12S, 16S	MH715304, MH715990
Gobiidae	*Gobiosoma longipala*	Twoscale Goby	12S, 16S	MH715305, MH715991
Gymnuridae	*Gymnura micrura*	Smooth Butterfly Ray	12S, 16S	MH715306, MH715992
Lutjanidae	*Lutjanus griseus*	Gray Snapper	12S	MH715307
Lutjanidae	*Lutjanus synagris*	Lane Snapper	12S, 16S	MH715308, MH715995
Mugilidae	*Mugil trichodon*	Fantail Mullet	12S, 16S	MH715311, MH715998
Rajidae	*Raja eglanteria*	Clearnose Skate	16S	MH715999
Sciaenidae	*Bairdiella chrysoura*	Silver Perch	12S, 16S	MH715297, MH715980
Sciaenidae	*Leiostomus xanthurus*	Spot	16S	MH715994
Sciaenidae	*Menticirrhus littoralis*	Gulf Kingfish	12S, 16S	MH715309, MH715996
Sciaenidae	*Micropogonias undulatus*	Atlantic Croaker	12S, 16S	MH715310, MH715997
Scorpaenidae	*Scorpaena brasiliensis*	Barbfish	12S, 16S	MH715312, MH716000
Sparidae	*Lagodon rhomboides*	Pinfish	16S	MH715993
Synodontidae	*Synodus foetens*	Inshore Lizardfish	16S	MH716002

Supplementary Table S3. Analyzed sequence reads (SE: ± 39647) of 18S rRNA gene and relative abundance (SE: ± 7.0%) of host sequences.

Sample	Analyzed sequence reads	Smalltooth sawfish sequences (%)
SF1*	29493	57.2
SF3	23835	58.0
SF4	28667	88.9
SF5	20692	50.9
SF6*	28434	99.4
SF7	24764	70.0
SF8	33341	87.5
SF9	28583	74.5
SF10	29794	51.8
SF11	38248	97.7
SF12	334831	99.8
SF13	421995	86.9
SF14	156696	15.7
SF15	26773	22.0
SF16	436500	91.3
Total	1662646	
Mean	110843	70.1

*Sample obtained from necropsy

Supplementary Table S4. Mitochondrial 12S and 16S rRNA gene species-signature sequences identified for analysis, with those resulting from our sequencing effort denoted by bold text. Data are in alphabetical order by family. Mean = 40.0 bp; Min = 36 bp; Max = 44 bp.

Family	Species	Common name	Gene	GenBank Accession No.	Species specific sequence	Base pairs
Achiridae	*Achirus lineatus*	Lined Sole	12S	NC_023768	CTACAACAGCATAGCACGAATTTTGTTTTGAAACATACAA	40
			16S	NC_023768	CTCCCGGATTCTTCCCTCAAGGACATTTAATTAATATATA	40
	Gymnachirus melas	Naked Sole	16S	JQ939050	CTCCCGGCTTCTTCCCAGACCGAGAGAGTTAGTCAAAAGG	40
	Trinectes maculatus	Hogchoker	12S	NC_023769	CTACCACAGCACACTACGAATGTTATGTTGAAACACACAA	40
			16S	NC_023769	CTCCCGGCTTCTTTCCTCACCACCACTTCAACACACCCCA	40
Acipenseridae	*Acipenser oxyrinchus*	Atlantic Sturgeon	12S	KP997217	CTGATACAGAAAATACACGAATAACACTGTGAAACCAGTG	40
			16S	KP997217	CTTAAGGCTTCTACTGCCACCCAGGTTATTACCAACAAAG	40
Albulidae	*Albula vulpes*	Bonefish	12S	X99180	CTGGTTCAGAATATTACGGATAATGCTTTGAAACAAGCAT	40
Amiidae	*Amia calva*	Bowfin	12S	NC_004742	CTAACTACAGAATACTACGGATGATTCTATGAAACATAGA	40
			16S	NC_004742	TTTAAGGATACTCAAACCAACTACAGTGTTACCACCAAAG	40
Anguillidae	*Anguilla rostrata*	American Eel	16S	KJ564271	CTTATGTAATTCTATAATCAAAACATTACCGACCAAAAGA	40
Aphredoderidae	*Aphredoderus sayanus*	Pirate Perch	12S	NC_004372	CTCAATGAGATTATTACTAATGGTAAATTGAAATATTAAC	40
			16S	NC_004372	CCTTTAATCTCTTAACCCCTAGACGATAATCCCCAACAGG	40
Rivulidae	*Kryptolebias marmoratus*	Mangrove Rivulus	12S	AF283503	CTACACAGGGAACCACGGATAGTACAATGAAATGTGTACA	40
			16S	AF283503	TATTCTTTAATCAAAACTAATTTAATTTTAACTAGACAAC	40
Ariidae	*Ariopsis felis*	Hardhead Catfish	12S	DQ990564	CTATAATAATAGAATACTACGGACGGCACTCTGAAATCAG	40
	Bagre marinus	Gafftopsail Catfish	12S	JX899751	CTATAATAATAGAATATTACGGACGGCACCCTGAAACCAG	40
			16S	JX899751	CTCACACTCCTTGACTCACAACTATTTATTAATATATGAA	40
Atherinidae	*Atherinomorus stipes*	Hardhead Silverside	12S	AF150001	CTAACCCAGGTAATACGAAAAATGCAATGAAAGCGTTAGA	40
Atherinopsidae	*Labidesthes sicculus*	Brook Silverside	16S	AY655511	CCCTAGACTTCTATAGTTTCGACTGGTACAACCTTTTCCC	40
	Menidia beryllina	Inland Silverside	12S	KX686069	CTGACCCAGGAAATCACGGACAGGGCCATGAAATTGGGCC	40
Balistidae	*Balistes capriscus*	Gray Triggerfish	12S	AY700238	CTGCCCCAGGTAACCACGGACGATTTATTGAAAAATAAAT	40
**Belonidae**	*Strongylura marina*	Atlantic Needlefish	12S	AF231554	ACTACTAATAAGCAGAATTGGCACAGCCCAAAACGTCAGG	40
	Strongylura notata	**Redfin Needlefish**	12S	AF150002	CTAACTCAGGGTATACGAAAAATGTCATGAAAAACACAT	39
			**16S**	**MH716001**	**CTATTAACTTTTTTACCCTTAATTGGTCTCACCCTTAAAC**	**40**
	Strongylura timucu	Timucú	12S	AF231570	CTAACCCAGGTAATACGAAAAATGCAATGAAAAAAGCAT	39

Family	Species	Common name	Gene	GenBank Accession No.	Species specific sequence	Base pairs
	Tylosurus crocodilus	Houndfish	12S	AF231578	CTAAACCAGGGTATACGGAAAATACAATGAAATACGTAT	39
Blenniidae	*Chasmodes saburrae*	Florida Blenny	12S	GQ865562	ACCCTCGTTAAACCTCACCCTTTTCTTGCCTATTCCCGCC	40
	Hypsoblennius hentz	Feather Blenny	12S	GQ865559	CTGAAATCAGGATATACGAATAATGTTTTGAAAAAGGCAC	40
Bothidae	*Bothus robinsi*	Twospot Flounder	12S	AF488509	GAGAAGGACAAGTCGTGTGCAGAATTGGCAAAGCCCAAGA	40
			16S	JQ939056	CCCCTAGTTTTTTCCGCACCCCAGGGGAGTTATTCACAGG	40
Callichthyidae	*Hoplosternum littorale*	Brown Hoplo	12S	HM114386	GCTACATTTTCTATTACAGAATATTACGAATAGCACTATG	40
Carangidae	*Caranx hippos*	Crevalle Jack	16S	DQ533167	CTCATGGCTTCTCCCTTCATATAATAATAACCACCTAATG	40
	Chloroscombrus chrysurus	Atlantic Bumper	12S	AY141387	CTACCCCTAGCGAACACGAATGATGCATTGAAACATGCAG	40
	Hemicaranx amblyrhynchus	Bluntnose Jack	16S	JQ939028	CTCATAGCTTCTCCTTAAACTGTGAGAGTTAGTCAAAGGG	40
	Trachinotus carolinus	Florida Pompano	12S	NC_024184	CTAAGATAGCGAACACGAATGATGCATTGAAACATACAAC	40
			16S	NC_024184	CTCCCGGGTTCTTCCTTCACCCCAATATGGTTTTTAACCA	40
	Trachinotus falcatus	Permit	16S	JQ939038	CTCCCGGCTTCTCCCTAAACCGCGAGAGTTAGTCAAAGGA	40
Carcharhinidae	*Carcharhinus acronotus*	Blacknose Shark	12S	NC_024055	TGAAGGACCAAAAGTAAGCAAAAAGAATTAAACTCCAAAA	40
			16S	NC_024055	GCATTACAGAGCCAACCCGTCTCTGTGGCAAAAGAGTGGG	40
	Carcharhinus brevipinna	Spinner Shark	12S	NC_027081	AGAGGAGATCAGAGTACTCCTCTGAAACTGGCTCTGGGAT	40
	Carcharhinus isodon	Finetooth Shark	16S	AY830729	TTCACCATCTAAACAAGACTTACTCGTCAAAGAAATATAT	40
	Carcharhinus leucas	Bull Shark	12S	NC_023522	CACCCTGTGAAGGATCAAAAGTAAGCAAAAAGAATT	36
			16S	NC_023522	TTTTTCACTATCTAGACAAGACTTACTCGTCAAAGAATTC	40
	Carcharhinus limbatus	Blacktip Shark	16S	AY830732	TTTTTTACCATCTAAACAAGATTTTCTTGTTAAAGAAACC	40
	Galeocerdo cuvier	Tiger Shark	12S	NC_022193	CCTGTGAAGGACTAAAAGTAAGCAAAAAGAATAAAACTTC	40
			16S	NC_022193	ATTTTTAATATTTAAACAAGACTTACTCGTTAAAAGACCT	40
	Negaprion brevirostris	Lemon Shark	12S	AY830756	TTCCTAAAAATATACGAATGGTGAACTGAAAACACACCTA	40
			16S	AY830756	ATTTTTTATTATCTAAACAAGACTTACTCGTCAAAGAAAC	40
	Rhizoprionodon terraenovae	Atlantic Sharpnose Shark	12S	AY830764	TTTTCAAAAATAAACGAATGGTAAACTGAAAAAACACCTA	40
			16S	AY830764	ATTTTTTACTACCTAAACAAGATTTACTCGTCAAAGGAAT	40
Catostomidae	*Erimyzon sucetta*	Lake Chubsucker	12S	KM273816	CTATTCCAGAATAACACGAACAGCACTATGAAAAAATGCT	40
			16S	KM282468	GGTGAGCTACCCCGAGACAGCCTACATAGGGCCAACCCGT	40
Centrarchidae	*Enneacanthus gloriosus*	Bluespotted Sunfish	16S	AY742519	CTCCCGGCTTCTTTTCTCATCTTAGTCTTTTCTCCCTACA	40
	Lepomis gulosus	Warmouth	12S	KM273835	TTAATAGAAAGTACACGAATGACTGACTGAAACGTCTTTC	40

Family	Species	Common name	Gene	GenBank Accession No.	Species specific sequence	Base pairs
			16S	AY742525	CTCCCGGCTTCTTACCTCAAACCCGTCGTAACCCCTTACA	40
	Lepomis macrochirus	Bluegill	12S	JN389795	TTAATAAAAAGAATACGAATGACTGACTGAAACGTCTTTC	40
			16S	JN389795	CTCTCGGATTCTTACCTCACTCTCGTCTAAACACCCAACA	40
	Lepomis marginatus	Dollar Sunfish	16S	AY742531	CTCTCGGGTTCTTATTTCATCTCCGCCTAAGCACCAAGCA	40
	Lepomis microlophus	Redear Sunfish	16S	AY742535	CTCCCGGCTTCTTATTTCATGTCCGTCTTTGCACCCAACA	40
	Lepomis punctatus	Spotted Sunfish	12S	MF621732	TTAACAAAAAGAATACGAATGATTGACTGAAATGTCTTTC	40
			16S	MF621732	CTCCCGGCTTCTTATTTCATATTCGTCTTCACACCCAACA	40
	Micropterus salmoides	Largemouth Bass	12S	NC_014686	CAGGGAATACGAACGATAAACTGAAATGTTTTTCCAAAGG	40
			16S	NC_014686	CTCGCAGCTTCTTCACTCACCCCTGTCCTACTCCCTACAG	40
	Pomoxis nigromaculatus	Black Crappie	12S	NC_028298	TACATTAGGGAATACGAACGACGTACTGAAATCTTACGTC	40
			16S	NC_028298	CTCCCGGCTTCTTTTCTCACCCTTGTCCTAACACCCTATA	40
Centropomidae	*Centropomus undecimalis*	Common Snook	12S	KC441979	CTACCACCAGCGCATACGAATAATGTATTGAAACATACTA	40
			16S	HQ731428	CCCCCGGCTTCTACATTCACTGACATCATAGTACATGATC	40
Clariidae	*Clarias batrachus*	Walking Catfish	12S	KC572134	CTACATCAGAATATTACGAACGGCACCCTGAAAAAGTGCC	40
			16S	KC572134	CTCGCACTCCTCAACTCAAAAACACACGTTAAAACACGAA	40
Clupeidae	*Brevoortia patronus*	Gulf Menhaden	16S	DQ912068	CCCGCCGCGCCCTCGCCCACAACAGTTTACTTAAACAAG	40
	Dorosoma cepedianum	Gizzard Shad	12S	NC_008107	CTGAAGCAGATTATTCACGAAAAGTCATCTGAAATCGATG	40
			16S	NC_008107	CCCGTTTTACCCTTGCCCACCGCAGTCTTACTAAAATTAG	40
	Dorosoma petenense	Threadfin Shad	16S	NC_009580	CCCGTTTTACCCTCATCCGCCATAGTTTTACTCAAATTAG	40
	Harengula jaguana	Scaled Sardine	12S	NC_016667	GAGCCAGATGACTACGGAGAGTCGCCTGAAACCTGGCGAT	40
			16S	NC_016667	CCCGAAAGCCCTAGCCCATTGCGGTCCCACCAAGATAAGG	40
	Opisthonema oglinum	Atlantic Thread Herring	12S	EU552701	CTGAAGCAGATTATTCACGGAAAGTCACCTGAAATTAGTG	40
			16S	DQ912074	CCCGTTTAACCCTTATCCACCGCAGTTTCACTAAAATTAG	40
	Sardinella aurita	Spanish Sardine	12S	EU552700	CACAACAAATATCCGCCTGGGAACTACGAGCGTTAGCTTA	40
			16S	DQ912067	CCCGCCGAACCCTCGTACACAACAGTTTTACTTAAATTAA	40
Coryphaenidae	*Coryphaena hippurus*	Dolphinfish	12S	KF814117	CTAACATAGTGAATACGAATAGTATATTGAAACATATACT	40
			16S	KF814117	CTCCCAGCTTCTCTCTTCACCTATTGTACACACAAAACAA	40
Cyprinidae	*Ctenopharyngodon idella*	Grass Carp	16S	NC_010288	CCTCGCACACCCCAAATCAAAAACATAACATCAAGACAAT	40
	Notemigonus crysoleucas	Golden Shiner	16S	NC_008646	CCTCGTATTACCCCAAACCAAAGAACATATCACTAAGGTA	40

Family	Species	Common name	Gene	GenBank Accession No.	Species specific sequence	Base pairs
	Opsopoeodus emiliae	Pugnose Minnow	12S	NC_033926	CTATTATAGAATATTACGAATATGTAACATGAAATAGTGC	40
			16S	NC_033926	CTCGTACCCCCTTAATCAAAAATATATTATTAAGACTACG	40
	Pteronotropis hypselopterus	Sailfin Shiner	12S	NC_033939	CTACTACAGAATACTACGGACATGCAACATGAAATAGTGC	40
			16S	NC_033939	AGCCTATATAATTTAGGGCTAACCCGTCTCTGTAGCAAAA	40
Cyprinodontidae	*Cyprinodon variegatus*	Sheepshead Minnow	12S	NC_028088	CTCATGGAGGAAATACGAATTGTGCCATGCAACAGCAC	38
			16S	NC_028088	ACCTTCTCCTATCAACCCTGGCCTGCCCTAAGCCCAGAAA	40
	Floridichthys carpio	Goldspotted Killifish	12S	AF449407	CTCTTCGAGGAAATACGGATTGTGCCATGAAACAGCAC	38
	Jordanella floridae	Flagfish	12S	NC_011387	CTCATTGAGGAAATACGAATTGTGCCATGCAACAGCAC	38
			16S	NC_011387	TCTTACCTTCTTCCCTCATCTCTGGCTTGCCCTAAACCCA	40
**Dasyatidae**	***Dasyatis americana***	**Southern Stingray**	**12S**	**MH715301**	**TTTCTAGAATACACGGACAGAAGCATGAAAAACTTCTTAA**	**40**
			**16S**	**MH715984**	**ATTAATTTTTCAACTCGATAACAAAAAATTCTTTTAACAA**	**40**
	Dasyatis sabina	**Atlantic Stingray**	**12S**	**MH715302**	**TTTCTAGAGTACACGAACAAAAGCATGAAAAACTTCTTGA**	**40**
			**16S**	**MH715985**	**ATTAATTTTTCAACTCGATAACAAAATCTTTTTTAACAAA**	**40**
Diodontidae	*Chilomycterus schoepfi*	Striped Burrfish	12S	AY700256	CTTCAACAGGGAACTACGAATGGCATTTTGAAATACATGC	40
	Diodon holocanthus	Balloonfish	12S	NC_009866	GCCCGCCCGGGAACTACGAGCACCAGCTTAAAACCCAAAG	40
			16S	NC_009866	TTTTGACTTCTTCCCCCAAAAACCTTAATTAAACCCACCA	40
Echeneidae	*Echeneis naucrates*	Sharksucker	16S	NC_022508	CCCTGTAATTTTCTTTCTTCATATATTTACTTCCTTAAAT	40
Elassomatidae	*Elassoma evergladei*	Everglades Pygmy Sunfish	12S	NC_003175	CTACTCAGCGAAAACGAACGACAAAATGAAATGTTTGTCT	40
			16S	NC_003175	CTTTTGGCTTCTCTAGTCATAGTAGATATTAGACCCCAAG	40
Eleotridae	*Dormitator maculatus*	Fat Sleeper	12S	KF415347	GACACAGTAAATACGAACGATGCGTTGAAACTAAGCATCT	40
			16S	KF415347	CTTTTGGTTTCTTTTGTCCAGCCTGGCTACGCCCACTTCA	40
Elopidae	*Elops saurus*	Ladyfish	12S	NC_005803	ACCAATAGTAAGCCTAATGAGCACAACTCAAAACGTCAGG	40
			16S	NC_005803	CCCACGACCTGCTTAAATCAAAAGAGCTAATTCTACCAAA	40
Engraulidae	*Anchoa hepsetus*	Striped Anchovy	12S	DQ912040	AAAAGTAAGCGAAATGGAAAATCTCCAGAACGTCAGGTCGA	41
			16S	DQ912075	CCTGCCTAGCCCAACTCACACCAGTTCTACAAAGAAAGAC	40
	Anchoa mitchilli	Bay Anchovy	12S	DQ912042	CACGAAGTGGGAAGAAATGGGCTACATTGCCTAATCTAGGCTAC	44
			16S	DQ912077	GTCAAGTTATAGCTGGTTGCCCAATAAATGAATACAAGTT	40
Ephippidae	*Chaetodipterus faber*	Atlantic Spadefish	12S	EF616894	CTGCCACAGAGAACACGCACGACATAATGAAAACAGATGT	40
			16S	DQ533172	CTCCCGGCTTCTTCCCTCCCTGGCACTCTGCCCCCCGCGA	40

Family	Species	Common name	Gene	GenBank Accession No.	Species specific sequence	Base pairs
Fundulidae	*Fundulus grandis*	Gulf Killifish	12S	NC_012377	CTGCTTAAGGAAATACGGATTGTGTTATGAAAAATCACAT	40
			16S	NC_012377	CTCTTGTCTTCTTATGCCAAATATGGATACCCAAACCTCA	40
	Fundulus majalis	Striped Killifish	12S	KX686099	CTGTCTAAGGAAATACGAATTGTATTCTGAAATGATTACA	40
**Gerreidae**	*Diapterus auratus*	Irish Pompano	12S	KJ468671	AACACCAGGGAATACGAATACTACGTTGAAACACGTACTT	40
	Eucinostomus argenteus	Spotfin Mojarra	12S	KJ622117	CTAATTCAGGGTATACGGATAATGAACTGAAACATTCATT	40
	Eucinostomus gula	**Silver Jenny**	12S	KT067840	CTAATACAGGGCACACGAACGATGACTTGAAATATACATT	40
			**16S**	**MH715986**	**AAGAGTTATTCAAAGGGGGGACAGCCCCTTTGAAAGAAAAA**	**41**
	Eucinostomus harengulus	**Tidewater Mojarra**	**12S**	**MH715303**	**CCGCCTATATACCGCCGTCGTAAGCTTACCCTGTGAAGGAA**	**41**
			**16S**	**MH715987**	**AAGAGTTATTCAAAGGGGGGACAGCCCCTTTGAAAGAAGAT**	**41**
	Eucinostomus jonesii	Slender Mojarra	12S	KT067842	CTAATATAGGGCACACGGATAATGCACTGAAACGTGCGTA	40
	Eugerres plumieri	**Striped Mojarra**	12S	KT067857	CTGCAATCAGGGAATTACGGATAACGCGTTGAAATACGTG	40
			**16S**	**MH715988**	**CTCTTGGCTTCTCTGCTCCGCGCCCTTCTACCCTGTCACCC**	**41**
	Gerres cinereus	Yellowfin Mojarra	12S	KY815278	CTATTATAGGGAACACGGACAACGTACTGAAATACGCGTT	40
			16S	JQ938957	CTCTCGGCTTCTTCCCAAACCTAGAGAGTTATTCAAAGGG	40
Ginglymostomatidae	*Ginglymostoma cirratum*	Nurse Shark	12S	NC_030189	TTTAACCAAAAAACACGGACAGTAAACTGAAAAATTACTT	40
			16S	NC_030189	ATTAATTTTTTATCACTAAACAAGACTATCTCATTAAAGT	40
Gobiesocidae	*Gobiesox strumosus*	Skilletfish	12S	KY656387	CTACTCAAGAGCACACGAAATAGGAATTGAAAAATCTCTT	40
			**16S**	**MH715989**	**CCCAGGCATCTACAACCCAAAAAAGGCCCCGCCTATTACA**	**40**
**Gobiidae**	***Bathygobius soporator***	**Frillfin Goby**	**12S**	**MH715298**	**CTGCCTCAGTACATACGGACGATGACCTGAAATTACATCCG**	**41**
			**16S**	**MH715981**	**CCTTTAATGTCTTAATTCCACCTCAGACTCGCTCACTTAAT**	**41**
	Ctenogobius boleosoma	**Darter Goby**	**12S**	**MH715299**	**CTATAAAGTGAGCAGAGTTGGCAATGCCCAAAACGCCAGGT**	**41**
			**16S**	**MH715982**	**TTACTCCAAGACAGCCTAATAATAGGGCAAACCCGTCTCTG**	**41**
	Ctenogobius smaragdus	**Emerald Goby**	**12S**	**MH715300**	**TTATAAAGTGAGCAGAGTTGGCAATGCCCAAAACGCCAGGT**	**41**
			**16S**	**MH715983**	**CTTTTGAGCTGCTCACCCCACCGTATTATTGTACCCCCAGG**	**41**
	Gobionellus oceanicus	**Highfin Goby**	**12S**	**MH715304**	**CCGCCTATATACCGCCGTCGTAAGCTTACCCTGTGAAGGAA**	**41**
			**16S**	**MH715990**	**CTCTTTAATTATTTTTTCTCAAGGCAAGGCCTAACAAGGAT**	**41**
	Gobiosoma bosc	Naked Goby	12S	AF491095	CTGTCCCAGTAAACACGGATCTTGCAATGAAACAAGCATT	40
			16S	AF491095	CTTTTGGATGCTCCCACCCCATAAAAACACACTTCTCAAC	40
	Gobiosoma longipala	**Twoscale Goby**	**12S**	**MH715305**	**CTGACACAGTGAAATACGAAAGATAGCTTGAAACAACCATC**	**41**

Family	Species	Common name	Gene	GenBank Accession No.	Species specific sequence	Base pairs
			**16S**	**MH715991**	**GAGCCCAGTTATAGCTGGTTGCCTGAGAAATGAATAGAAGT**	**41**
	Gobiosoma robustum	Code Goby	12S	AF491087	CTGTATCAGTAAACACGGATTTTGCACTGAAATAAGCATC	40
			16S	AF491087	CTTTTGGATGCTCCCACCTTCTAGAAATACCTTTTCTCCC	40
	Lophogobius cyprinoides	Crested Goby	12S	KF415410	CTGGTACAGTGAATTTACGAAACTTCATGCTCTGAAACAA	40
			16S	KF415410	CTTTTGAGCTGCTCACCCCACCGTATTATTTACCCCCAGG	40
	Microgobius gulosus	Clown Goby	12S	AF491111	CTGAATCAGTAAAAACGGATTTTGCACTGAAATGAGCATC	40
			16S	AF491111	CTTTTCATTTCTTCTACCCACCCGCACCCGCCCCCGGCTA	40
	Microgobius thalassinus	Green Goby	12S	AF491113	CTGAAACAGCAAACACGGATTATGCACTGAAATAAGCATC	40
			16S	AF491113	GGCAATACCAGCCTCCGGCTAAGAGAAATTTTAAGAGTTA	40
**Gymnuridae**	***Gymnura micrura***	**Smooth Butterfly Ray**	**12S**	**MH715306**	**ATCTGAAGGAGGATTTAGCAGTAAGAGGGGACTATGAGAG**	**40**
			**16S**	**MH715992**	**TTAATTCTTCAAGCAAGCAATAAAAAACTTTTAACACAAG**	**40**
Haemulidae	*Haemulon aurolineatum*	Tomtate	12S	EF095568	CTACTGCCAGTGAACACGAATAGTGCACTGAAATATGCAC	40
Hemiramphidae	*Hyporhamphus unifasciatus*	Atlantic Silverstripe Halfbeak	12S	AF231581	CTATCATAGGACATACGGATAATGTAATGAAACGTACATT	40
Ictaluridae	*Ameiurus catus*	White Catfish	12S	DQ421868	CTACAACTAGAATATTAAACGAATGGCACTATGAAAATTA	40
	Ameiurus natalis	Yellow Bullhead	12S	MF621735	CTATAACTAGAATATTAAACGAACGGCACTATGAAAATTA	40
			16S	MF621735	CCCTCACCTCACAAACACCTTTAAAATACACGAGCCCGAG	40
	Ameiurus nebulosus	Brown Bullhead	12S	MF621733	CTACAACTAGAATATTAAACGAATGGCATTATGAAAATTA	40
			16S	MF621733	CCCTCACCTCACCAACACCTTTATACTACACGAGCCCGAG	40
	Ictalurus punctatus	Channel Catfish	12S	NC_003489	ACCTAGAATATTACGAATGGCACCATGAAAATAATGCCTG	40
			16S	NC_003489	CTCGTACTCCTCATCTCACAAATACTTTTATATTAACGAG	40
	Noturus gyrinus	Tadpole Madtom	12S	AY458874	CTACATCTAGAACATAAACGAATGGCGCCATGAAAATGCT	40
			16S	AY458874	CTCGCACTCCTCACCTCACAACACATAATTCTACGAATAT	40
Kyphosidae	*Kyphosus saltatrix*	Bermuda Chub	12S	KC136572	CTAACACAGTGAATTACGGACGATATACTGAAATTTATAT	40
			16S	KC136572	CTCCCCTCACCCTCGTCCGACCCCCCTCTGACACCTTAAG	40
Labridae	*Halichoeres bivittatus*	Slippery Dick	12S	AY279599	GTAATTATACTAAATACGGATGACATTCTGAAAAAATATC	40
	Lachnolaimus maximus	Hogfish	12S	AY279618	ATACCACATTGAATACGAATAGTAGAATGAAACATCTACT	40
Lepisosteidae	*Lepisosteus platyrhincus*	Florida Gar	12S	NC_029715	CTATGTCAGAATATTACGAACAACACCATGAAATTGGTAT	40
			16S	NC_029715	CTCAAGACATGCTCCTAACCGCCCCAAGTACTTACTTCAC	40
Lobotidae	*Lobotes surinamensis*	Atlantic Tripletail	12S	NC_026233	CTATTTCAGAGAATACGGATGGTGTAATGAAACTTACACA	40

Family	Species	Common name	Gene	GenBank Accession No.	Species specific sequence	Base pairs
			16S	NC_026233	CTTTGGGCTTCTTTACTCACTAAGCATCTTACAATTCACG	40
Loricariidae	*Pterygoplichthys disjunctivus*	Vermiculated Sailfin Catfish	16S	NC_015747	CTTGCACTCCTCACCTCACAAAAAGTTACCACTAACCTAT	40
**Lutjanidae**	*Lutjanus analis*	Mutton Snapper	12S	EF095569	CTAATATAGTGTAATACGAACGATACACTGAAATACGTAT	40
	Lutjanus griseus	**Gray Snapper**	**12S**	**MH715307**	**TCTCAAGAATATACGAACAGAAATATGAAAAATTTCTTAA**	**40**
			16S	JQ938964	CTCCCGGCTTCTTTCTAAACCGAGAGAGTTAATCAAAGGG	40
	Lutjanus synagris	**Lane Snapper**	**12S**	**MH715308**	**TAACATAGTGAAATACGAACGATGCACTGAAATACGCATC**	**40**
			**16S**	**MH715995**	**CTCCCGGCTTCTTTCTTCACCCCTAGTCTTTACCCCCTTCT**	**41**
Megalopidae	*Megalops atlanticus*	Tarpon	12S	NC_005804	CCCTCTCGAGTACACGAATAGAAACATGAAAAACTTCTTA	40
			16S	NC_005804	CTCGAAGCCTGCCTAAACCAAACACCAACAACACCAAAGA	40
Mobulidae	*Manta birostris*	Giant Manta	12S	KF413894	CTCAACCCAGAATACTTGCGAACGACACAGTGAAACCTGA	40
			16S	KF413894	ATTAACTTTTCAATCAGGTAATAAAAAATTTTTAACTAAA	40
Monacanthidae	*Aluterus schoepfi*	Orange Filefish	12S	KT600932	CTACTTAGAGAACACGAATGATAAACTGAAAAACGTATCT	40
	Stephanolepis hispidus	Planehead Filefish	12S	KT600974	TTTACAAAAACAATACGAACGGTAAACTGAAAAACACCTA	40
**Mugilidae**	*Mugil cephalus*	Striped Mullet	12S	KP018403	CTTTGATAATTTATTACACCCATTATCCGCCAGGGAACTA	40
			16S	KP018403	CTAGTAATAGTCAAGCAAAAAGCATTTTAGTTTGACCCCC	40
	Mugil curema	White Mullet	12S	NC_017889	TTTGATAGTTCAACCACGCCCACTATCCGCCTGGGTACTA	40
			16S	KF375100	ACAACTTTCCCAGGAGGGTAAAGATCATATTTCATGAAGG	40
	Mugil trichodon	**Fantail Mullet**	**12S**	**MH715311**	**CTAATACAGAGAATACGAATGATGAACTGAAATGTACATCT**	**41**
			**16S**	**MH715998**	**TTCTTATATTCTTCCCCCCTTTCGGTCATCATGGCCCTATC**	**41**
Mullidae	*Mullus auratus*	Red Goatfish	16S	JQ938968	CCCACGGCTTCTCCTTATACCGCGGGAGTTAGTCAAAGGG	40
Muraenidae	*Gymnothorax saxicola*	Honeycomb Moray	12S	JX242931	CTGACCCAGAAATATCACGAACAGTGCAATGAAATACGCA	40
Myliobatidae	*Aetobatus narinari*	Spotted Eagle Ray	12S	JX978310	TCTCTATCAAGAACACACGAAAAGAAGCATGAAAACCCTC	40
Narcinidae	*Narcine bancroftii*	Lesser Electric Ray	12S	NC_034772	GAATAACAGTAAGCAAAATGAATAAAATATTCAATACGTC	40
			16S	NC_034772	ATTAATTATTCAATTACGTACCATATAAGAATCTTAGACC	40
Ophichthidae	*Ophichthus gomesii*	Shrimp Eel	12S	AY430249	CTGAAACAGAATACTACGAAAGGTGCCATGAAACAAACAC	40
Ostraciidae	*Acanthostracion quadricornis*	Scrawled Cowfish	12S	AY700275	CTTTTCCAGTGAAATACGAACGATGTACTGAAATATACAT	40
Paralichthyidae	*Anclyopsetta quadrocellata*	Ocellated Flounder	16S	JQ939064	CCCCTAATTTCCCCACAAACTAGGAGTGTAACTCAAAGGG	40
	Etropus crossotus	Fringed Flounder	16S	JQ939068	CCCTTGGTTTTTCCCGAAACCAAGGGAGTTATTCATAGGG	40
	Paralichthys albigutta	Gulf Flounder	12S	AF316017	TAGAACTAGCAAATACGAATGATACATTGAAATATGT	37

Family	Species	Common name	Gene	GenBank Accession No.	Species specific sequence	Base pairs
			16S	JQ939111	CCCCTGGGTTCCCCACAAACCAAGGGTGTTAGTCAAAGGG	40
	Paralichthys lethostigma	Southern Flounder	12S	NC_029223	CTAAGACTAGCAAATACGAACGATGCATTGAAATATGCAA	40
			16S	NC_029223	CCCCTGGGTTCCCGACTCATTCACTGTTATCACCCTTCAG	40
	Syacium papillosum	Dusky Flounder	12S	AF488503	CTAATTCAGGGCACACGAAAAATATAATGAAAAACGTAT	39
Phycidae	*Urophycis floridana*	Southern Hake	12S	FJ215094	CTGCCCCAGAGAACTTATACGGATGGTATATTGAAATTAA	40
			16S	FJ215198	ATAGCTAAAGGAGTTAGTCAGAAAGGGTACAGCTTTTCTG	40
Poeciliidae	*Gambusia holbrooki*	Eastern Mosquitofish	12S	NC_028274	CTTCCCCCAGGAAACACGAATTGTGCTATGAAATAACACA	40
			16S	NC_028274	CTTTAATTATCTCGTACCCCCGACGGCCTAACCAACAGCC	40
	Heterandria formosa	Least Killifish	12S	EF017473	CTCTCCTAGGAAAAACGAATTGTGTCATGAAACAACACAT	40
	Poecilia latipinna	Sailfin Molly	16S	NC_035305	CTCTACTTATCTCCCACCCCAATAGTCCCACCACAAACCT	40
Polynemidae	*Polydactylus octonemus*	Atlantic Threadfin	16S	JQ938973	GATAGGAGTTCAGCCTTAAAGATTCTTTTGAAACTTTAAG	40
Pomacentridae	*Abudefduf saxatilis*	Sergeant Major	12S	FJ616293	CTAAACCAGGAAATACGGACAATGTATTGAAACGTACATT	40
Pomatomidae	*Pomatomus saltatrix*	Bluefish	12S	NC_022507	CTAATGAAGCGAACACGAATGGTGCACTGAAAACTGTGCG	40
			16S	NC_022507	CTTTGGGCTTCTCTCTTCACTACAATTTATACTTATTACT	40
Pristidae	*Pristis pectinata*	Smalltooth Sawfish	12S	NC_027182	CTTTTAAGAAAAAACGAACAGTACAATGAAAAATTACTCA	40
			16S	NC_027182	ATTAATTTATTCTACCTGCCACAAATGGAACATTCTCATA	40
Rachycentridae	*Rachycentron canadum*	Cobia	12S	NC_011219	CTAAAAATAGTGAATACGAATAATACATTGAAACATGTAT	40
			16S	NC_011219	CTCCTGGCTTCTCAATTCATCTCAAAACAAATGACTCAAC	40
**Rajidae**	***Raja eglanteria***	**Clearnose Skate**	12S	KF317714	CACAAAAGTAAGCATAACGGATTTCCTCCAAAACGTCAGG	40
			**16S**	**MH715999**	**ATAATCCTTCACCTCGCCCACCAAGATTACCAAAACAAGG**	**40**
Rhinobatidae	*Rhinobatos lentiginosus*	Atlantic Guitarfish	12S	AY830717	CTATTAAGAAAAAACGGATATTATAATGAAAAACTACTTA	40
			16S	AY830717	ATTAATTTACTCCAACTTGCCATAAATATTACTTATATAT	40
Rhinopteridae	*Rhinoptera bonasus*	Cownose Ray	12S	JX241047	CTCTTTAGAGTATACGGATAGAAACATGAAAATCTTCTTA	40
Scaridae	*Nicholsina usta*	Emerald Parrotfish	12S	AY279624	ATGGAACAATGAATACGAATGGTTAATTGAAAGATTTTCC	40
**Sciaenidae**	***Bairdiella chrysoura***	**Silver Perch**	**12S**	**MH715297**	**CTAATGCAGAGGAAACGAATGATGTGCTGAAACGCACATC**	**40**
			**16S**	**MH715980**	**CTTCCGGCTTCTCCCCTCACACCTGTCCTCCTTCCCCTTAG**	**41**
	Cynoscion arenarius	Sand Seatrout	12S	AF128401	CTGAAATATATATCTGAAGGAGGATTTTAGCGGTAAGCAT	40
			16S	AF128401	TCTCCCCTCACTCCTGTTTTTCTCCCCATGATAACCAGAT	40
	Cynoscion nebulosus	Spotted Seatrout	12S	AF128402	CTAACATAGAGAAAACGAATGATATACTGAAACATATATC	40

Family	Species	Common name	Gene	GenBank Accession No.	Species specific sequence	Base pairs
			16S	AF128402	TCTCCCCTCACCCCCGTTTTCTCGCCATGACAACCAAAT	40
	Leiostomus xanthurus	**Spot**	12S	KX686090	CTAATACAGAGAAAACGAATGATGTACTGAAATACACGTC	40
			**16S**	**MH715994**	**CTACTCCAAGACAGCCTAATAATAGGGCAAACCCGTCTCTG**	**41**
	Menticirrhus americanus	Southern Kingfish	12S	DQ874712	AGAAATGGGCTACATTCTCTCGAGCACTGAAAGAAGGAGG	40
	Menticirrhus littoralis	**Gulf Kingfish**	**12S**	**MH715309**	**TAATATAGTGAAATACGAACGATACACTGAAATACGTATC**	**40**
			**16S**	**MH715996**	**TCCCGGATTTCTCCCGTCCATCCCAGTTTATACCCCTGCA**	**40**
	Menticirrhus saxatilis	Northern Kingfish	12S	KX686072	AGAAATGGGCTACATTCTCTAACCTCAGAGAAAACAAATG	40
	Micropogonias undulatus	**Atlantic Croaker**	**12S**	**MH715310**	**CTAATCTCAGAGAAAACGAATGATGCACTGAAACACGCATC**	**41**
			**16S**	**MH715997**	**CTCTCGGATTCTTCCCTCACCACTGTTCTCCTCCCCCTTGA**	**41**
	Sciaenops ocellatus	Red Drum	12S	NC_016867	CTAATACAGAGAATACGAATGATGTACTGAAATACACATC	40
			16S	NC_016867	CTCTCGGATTCTTCCCTCACCCCTGTCTTCCTCTCCCCGA	40
Scombridae	*Euthynnus alletteratus*	Little Tunny	12S	NC_004530	CTACTATTAGCGAATACGAACGATGCACTGAAAACGTTCA	40
		Little Tunny	16S	NC_004530	TTCGCCATGGTCTTACCCCTACCGATACTCAAAAGAAGTC	40
	Scomberomorus cavalla	King Mackerel	12S	NC_008109	CTATTCTAGCGAATACGAACGATAGTACTGAAAACGTACA	40
			16S	NC_008109	CTCCAGGCTTCTCTCTTCACCGCGGTTTAACCCCTGCCGA	40
	Scomberomorus maculatus	Spanish Mackerel	12S	DQ874689	CCCGTCGGTAAATACGAATGGTTTATTGAAAAATAAAACT	40
**Scorpaenidae**	***Scorpaena brasiliensis***	**Barbfish**	**12S**	**MH715312**	**TGAAATAGAGCATACGAACGATGAACTGAAAACTCATCTG**	**40**
			**16S**	**MH716000**	**TTTCAGATTCTTTTTTTAATTAAGTAATTCACTTCTAAAA**	**40**
Serranidae	*Centropristis striata*	Black Sea Base	12S	KX686079	CTATGATAGTGAATACGAATGATATCCTGAAACGGACATC	40
	Diplectrum formosum	Sand Perch	16S	FJ548768	CTCCCGGCTTCTTAATTCCCATAATAAACCTTATCTATAG	40
	Epinephelus itajara	Atlantic Goliath Grouper	16S	FJ548765	TTCTTCCTTCTCCAACCCCCTCTGTCTCAACAGATATTTT	40
**Sparidae**	*Lagodon rhomboides*	Pinfish	12S	KX686070	CTTATACACAGGGAACTACGAATGGTATACTGAAAACGTA	40
			**16S**	**MH715993**	**TCCCGGCTTCTCACCTCAATTTTGGTTTATACTTCAATTA**	**40**
Sphyraenidae	*Sphyraena barracuda*	Great Barracuda	12S	NC_022484	GCAAATAGTAGGCAAAGTTGGCACAGCCCAAAACGTCAGG	40
			16S	NC_022484	CTCCCGGCTTCTCTCTTCACACTTGCACCAATGCTTTAAT	40
	Sphyrna lewini	Scalloped Hammerhead	12S	NC_022679	CTAACATTAGCGAATACGAACGCTGCATTGAAATATGCAA	40
			16S	NC_022679	ATTAATTTTTTATTACCCAAACAAGACTTCCTTATTAAAG	40
	Sphyrna mokarran	Great Hammerhead	12S	NC_035491	TTTACAAAAACATATACGAATGGTAGACTGAAAAACACCT	40
			16S	NC_035491	TTTTTATTATCTAAACAAGACTTTCTCGTCAAAGAAACCC	40

Family	Species	Common name	Gene	GenBank Accession No.	Species specific sequence	Base pairs
	Sphyrna tiburo	Bonnethead	12S	NC_028508	TTTTATTAAAACATACGAACGGTAAACTGAAAAAATACCT	40
			16S	NC_028508	TTTTTTTTACCTAGACAAGACTTCCTTATTAAAGGAATCC	40
Stromateidae	*Peprilus burti*	Gulf Butterfish	12S	AP012947	CTAGCTTAGCGAATACGAACGATGTAATGAAAATGTACAT	40
			16S	AP012947	CTCCCGGTTTCTCCCTTCACCACGGTCATACCCCTACCGA	40
Syngnathidae	*Anarchopterus criniger*	Fringed Pipefish	12S	KY065791	TCCATCGATGAACAACGAATGATTTATTGAAAAATAAACC	40
	Hippocampus erectus	Lined Seahorse	12S	NC_022722	AGAACAACAGTGAGCAGAACTAGTAATAACCCAAAACGTC	40
			16S	NC_022722	CCATATTATACCCTAAAACTCATACAAATTAAAATAAATA	40
	Hippocampus zosterae	Dwarf Seahorse	12S	KY065837	TTTACTAATGAATACGGATGGCATATTGAAACATAAACCT	40
	Microphis brachyurus	Opossum Pipefish	12S	NC_010273	TTTACCAACGCATAACGGACAACATGTTGAAACCACATAT	40
		Opossum Pipefish	16S	NC_010273	CTGATTTTACCCTCAAGCATTACTAAATTGTATTAAAGCC	40
	Syngnathus floridae	Dusky Pipefish	12S	AF354957	GCTATTAAAGCACACACGGAACTCGTATTGAAACATACGT	40
	Syngnathus louisianae	Chain Pipefish	12S	KY065876	TCAACCGATAAACACGGATGATTAATTGAAAAATGAATCT	40
	Syngnathus scovelli	Gulf Pipefish	12S	AF354970	TTTGTCAACCGATAAACACGGATGATTGATTGAAAAATAA	40
**Synodontidae**	***Synodus foetens***	**Inshore Lizardfish**	12S	KC441961	CTTTCCGCAGAGAAAAACGGAAGGGGCAGTGAAACCTGCC	40
			**16S**	**MH716002**	**CCTTCCCCATTTTTACTCAAATTCTAATACCCATCAGACA**	**40**
Tetraodontidae	*Lagocephalus laevigatus*	Smooth Puffer	12S	NC_015345	GGCTACATTCTCTGCCCAGAGAACAACGAATGATGTGTTG	40
			16S	NC_015345	TTTTTGGCTTCTTGCCTCACAAATAGTATTAATAAAACAG	40
	Sphoeroides spengleri	Bandtail Puffer	12S	AY700284	AGAGATGGGCTACATTCGCTCGAGTACTGAAAGAAGGAGG	40
Triakidae	*Mustelus norrisi*	Florida Smoothhound	12S	AY830755	TTTACCAAAAAACACGAATGGTGAACTGAAAACACGCCTA	40
			16S	AY830755	ATTAATTCTTTATTATCTAGACAAGAATTTCTCGTCAAAG	40

Supplementary Table S5. Mitochondrial 12S and 16S rRNA gene sequence read information of successful identifications using the species-signature sequence method.

Sample	Analyzed sequence reads		Sequences identified by this method				OTUs identified by this method	
			12S		16S			
	12S	16S	No.	%	No.	%	12S	16S
SF1	5456	21202	75	1.4	0	0	1	0
SF3	2993	3026	571	19.9	0	0	16	0
SF4	ND*	16344	ND	ND	0	0	ND	0
SF5	ND	14967	ND	ND	1712	12.3	ND	15
SF6	3675	24865	44	1.2	17	0.1	6	1
SF7	4199	11418	166	4.2	335	3.0	5	7
SF8	5958	12732	486	8.5	30	0.2	19	1
SF9	4783	8607	1458	31.0	28	0.3	3	1
SF10	7300	9213	1668	24.2	2032	22.4	17	8
SF11	9702	7080	428	4.4	0	0	3	0
SF12	7371	8584	217	3.0	0	0	4	0
SF13	16437	10169	231	1.4	12	0.1	7	1
SF14	4945	9795	1315	29.4	248	2.8	32	7
SF15	10896	4248	10698	98.5	18	0.4	4	1
SF16	933	4069	0	0	20	0.5	0	1
Total	84648	166319						
Mean	6511	11088	1335	17.4	297	2.8	9	3
± SE	± 1112	± 1593	± 796	± 7.5	± 168	± 1.6	± 3	± 1

*ND = no data

Supplementary Table S6. Relative taxonomic abundance of mitochondrial 12S and 16S rRNA gene sequences (%) in smalltooth sawfish fecal samples after removal of host sequences.

	SF1		SF3		SF4		SF5		SF6		SF7		SF8		SF9		SF10		SF11		SF12		SF13		SF14		SF15		SF16	
Species	12S	16S	12S	16S	12S*	16S	12S*	16S	12S	16S	12S	16S	12S	16S	12S	16S	12S	16S	12S	16S	12S	16S	12S	16S	12S	16S	12S	16S	12S	16S
Anchoa hepsetus									30.9	21.2																				
Anchoa mitchilli									56.9		1.0																100.0	100.0		
Bairdiella chrysoura			76.9								48.4				0.2		19.1		1.4		100.0		55.7		62.1	0.4				
Cynoscio arenarius								0.2			42.3	11.6			0.2	100.0														
Cynoscion nebulosus																	80.6	62.7												
Cynoscion sp.															85.1		0.1													
Dasyatis americana									12.2	78.8																				
Dorosoma cepedianum																									9.0	10.5				
Elops saurus													87.6	93.5															1.4	
Eucinostomus harengulus											2.6	83.2					0.2	37.3		89.9			1.6	100.0		10.8				3.8
Eugerres plumieri															14.5															
Lagodon rhomboides			23.1																				42.7							
Leiostomus xanthurus								20.7																	28.9	78.3			98.6	96.2
Menticirrhus sp.								2.7																						
Microgobius gulosus											5.7	5.2																		
Micropogonias undulatus								76.1											98.6	10.1										
Mugil cephalus	100.0	100.0																												
Strongylura notata													11.0	6.5																
Synodus foetens								0.3					1.4																	

*No data
